# An Overview of Current Research on Mesenchymal Stem Cell-Derived Extracellular Vesicles: A Bibliometric Analysis From 2009 to 2021

**DOI:** 10.3389/fbioe.2022.910812

**Published:** 2022-06-24

**Authors:** Xudong Zhang, Yimeng Lu, Shanshan Wu, Siwen Zhang, Shuyu Li, Jichun Tan

**Affiliations:** ^1^ Center of Reproductive Medicine, Department of Obstetrics and Gynecology, Shengjing Hospital of China Medical University, Shenyang, China; ^2^ Key Laboratory of Reproductive Dysfunction Disease and Fertility Remodeling of Liaoning Province, Shenyang, China

**Keywords:** mesenchymal stem cell-derived extracellular vesicles, bibliometric analysis, CiteSpace, VOSviewer, therapeutic

## Abstract

**Background:** Mesenchymal stem cell-derived extracellular vesicles (MSC-EVs) are important mediators of intercellular communication and participate in numerous physiological and pathological processes in the body. This study aims to introduce the research status, analyze the research hotspots, and predict the development trend through bibliometric analysis of MSC-EVs.

**Methods:** We searched all relevant literature on MSC-EVs from 2009 to 2021 in the Web of Science. R-bibliometrix, VOSviewer, and CiteSpace software were used to visualize the quantitative analysis of the published literature, including co-authorship, co-occurrence, citation, and co-citation, to provide objective presentation and predictions in the field.

**Results:** A total of 1595 articles and reviews on MSC-EVs published between 2009 and 2021 were identified. The annual publication outputs increased at an exponential rate, reaching as high as 555 publications in 2021. China contributed the most publications (*n* = 899, 56.36%) and had the most citations (*n* = 24,210). The United States had the strongest intensity of cooperation in this field. Shanghai Jiao Tong University had the maximum number of publications (*n* = 79). In terms of the number of publications and co-citations, the journal of Stem cell research & therapy ranked first. Camussi G was the most productive and most cited author. The top three themes in the research area were cell biology, research experimental medicine, and biochemistry molecular biology. Keyword co-occurrence and co-citation clustering analysis revealed that studies of MSC-EVs covered cellular origin (bone marrow mesenchymal stem cell, adipose-derived mesenchymal stem cell), injurious diseases (spinal cord injury, acute lung injury, ischemia/reperfusion injury, acute kidney injury, traumatic brain injury), tumor (breast cancer, tumor microenvironment), biological processes (drug delivery system, angiogenesis, inflammation, proliferation, differentiation, senescence), and molecular mechanisms (signaling pathway, signal transduction, oxidative stress, VEGF, TGF β).

**Conclusions:** Studies on MSC-EVs have shown a steep growth trend in recent years. Available studies mostly focused on the therapeutic effects and underlying mechanisms of MSC-EVs in aplastic diseases. Multidisciplinary integration is a development trend in this field, and senescence-related topics might be the focus of future research on MSC-EVs.

## 1 Introduction

Extracellular vesicles (EVs) are nanoscale particles detached from the cell membrane or secreted by cells and carry the proteins, RNA, DNA, and lipids of the derived cells. EVs can be classified in different populations based on their biogenetic pathway, composition, and physical characteristics, such as size or density, giving rise to three major categories: apoptotic bodies (50–2000 nm), microvesicles (50–1500 nm), and exosomes (50–120 nm) (Simon et al., 2018). EVs, as mediators of material and signal communication between cells, participate in various physiological and pathological processes in the organism (Nawaz et al., 2016). From gonogenesis, embryogenesis to age-related cellular senescence, EVs are involved in almost the entire life cycle of human beings (Machtinger et al., 2016; Takasugi, 2018). Moreover, EVs play an important role in the occurrence and development of various diseases, such as cancer (Willms et al., 2018), neurodegenerative diseases (Hill, 2019), cardiovascular diseases (Gaceb et al., 2014), metabolic diseases (Akbar et al., 2019), and musculoskeletal diseases (Murphy et al., 2019). To date, the application potential of EVs is mainly in the diagnosis and treatment of diseases. Based on the analysis of EVs, cell-specific or disease-specific proteins can be identified, thereby contributing to disease diagnosis (Schou et al., 2020). The therapeutic potential of EVs, on the one hand, is as a vehicle for drug delivery (Kalluri and LeBleu, 2020), and on the other hand, the specific cell-derived EVs themselves have therapeutic effects.

In the 1970s, Friedenstein’s research team extracted a type of spindle-shaped stromal cells from the bone marrow that can attach to plastic and differentiate into osteoblasts, chondrocytes, adipocytes, under corresponding *in vitro* conditions, and were subsequently defined as bone marrow-derived mesenchymal stem cells (BMSCs) (Väänänen, 2005). Mesenchymal stem cells (MSCs) are a class of adult stem cells originating from the mesoderm, with self-renewal and multi-directional differentiation potential (Li and Hua, 2017). MSCs can secrete a variety of cytokines and growth factors to regulate immunity, inhibit fibrosis and apoptosis, promote angiogenesis, activate endogenous stem/progenitor cells, and rebuild and maintain cellular microenvironmental niches (Andrzejewska et al., 2021). Furthermore, compared with embryonic stem cells, MSCs have the advantages of broad sources, easy access, low risk of tumorigenesis, and no ethical controversy (Li and Hua, 2017). Therefore, MSCs have become the main target of clinical translational research on stem cells.

In recent years, it has been demonstrated that the biological effects of MSCs largely depend on their secretome/EVs. After confirming that BMSCs and their conditioned medium can improve retinal ischemia/reperfusion injury, Mathew *et al.* further confirmed that EVs in the conditioned medium were the main components (Mathew et al., 2020). Zhang *et al.* compared the therapeutic effects of menstrual blood MSCs and small EVs derived from menstrual blood MSCs in the intrauterine adhesion (IUA) rat model, and no significant difference was detected between the two treatment methods. Specifically, both MSCs and small EVs could effectively repair endometrial damage, promote angiogenesis, and remodel fertility in IUA rats (Zhang S. et al., 2021). Mesenchymal stem cell-derived extracellular vesicles (MSC-EVs) can not only imitate the therapeutic effect of MSCs, but also have less risk, higher safety, and higher application value as a cell-free therapy. Moreover, MSC-EVs are not easily cleared by metabolism and can penetrate the blood-brain barrier due to their small size, which has advantages in the treatment of neurological diseases (Imafuku and Sjoqvist, 2021).

Bibliometric analysis is conducted to analyze the literature in the scientific field through qualitative and quantitative assessments, clarifies the development trends of scientific research objectively and visibly, and plays an important role in the current status of research and the prediction of the development direction (Brandt et al., 2019). In addition, it allows for the identification of collaborative relationships among authors, institutions, and countries, and the evaluation of the academic contribution of countries, institutions, journals, and authors in a specific field (Xia et al., 2022). Notably, the period of the included studies has a significant impact on the bibliometric results, therefore, timely updating is essential to grasp the research frontier. Recently, MSC-EVs have become a research hotspot, while the relevant bibliometric analysis studies are limited, mainly focusing on the EVs or exosomes without a specific source (Yiran et al., 2017; Wang et al., 2019), or the role of exosomes in a particular sort of disease, including cardiovascular disease, cancer (Ma et al., 2021; Shi et al., 2021; Teles et al., 2021). In this study, we performed a bibliometric analysis of literature on MSC-EVs published from 2009 to 2021, covering the number of publications, countries, institutions, authors, journals, and keywords, to summarise current thematic trends and hot topics and provide guidance for future research.

## 2 Methods

### 2.1 Data Acquisition and Retrieval Strategy

Web of Science (WoS), a world-recognized database, was used to search for publications related to MSC-EVs. On December 31, 2021, we input the search terms “mesenchymal stem cell-derived extracellular vesicle”, “mesenchymal stem cell-derived microvesicle”, “mesenchymal stem cell-derived exosome”, “mesenchymal stem cell-derived apoptotic body”, “extracellular vesicle derived from mesenchymal stem cell”, “microvesicle derived from mesenchymal stem cell”, “exosome derived from mesenchymal stem cell” and “apoptotic body derived from mesenchymal stem cell” in the WoS Core Collection including SCI-EXPANDED, with no restriction on language. The timespan was set from January 1, 2009, to December 31, 2021. A total of 2739 articles and reviews were obtained, and after excluding those on unrelated search topics (*n* = 1144), the remaining 1595 publications were used for subsequent analysis.

### 2.2 Bibliometric Analysis and Visualization

The analysis of annual production, the number of national, institutional, and journal publications, and the research areas were realized through the “Analyze Results” of WoS. The trend in publications over years was analyzed by the curve-fitting function of IBM SPSS Statistics 22.0 software (IBM Corp., Armonk, NY, United States). A two-tailed *p*-value < 0.05 was considered significant. Journal impact factors and category quartiles were acquired from the 2020 Journal Citation Reports (Clarivate Analytics, Philadelphia, United States). Bibliographic information for publications, including country distribution, year of publication, and citations, was automatically analyzed using the bibliometrix package in R 4.1.2. Statistical management of all data is carried out in Microsoft Excel 2016.

In this study, VOSviewer and CiteSpace are two key software for bibliometric analysis and visualization. WoS data were converted to txt format for further analysis. VOSviewer (1.6.17, Leiden University, the Netherlands) was employed to analyze citations of publications, co-authorship of countries/institutions/authors, co-citation of authors/journals/references, and co-occurrence of keywords. The VOSviewer parameter settings mainly included the counting method: full counting and item threshold, which was adjusted with different items. In the visual map, various nodes represent countries/institutions/journals/authors/references/keywords, the size of the nodes represents the number of publications/citations/frequency of occurrence, different colors represent clusters/the average appearing year, and the thickness of the line represents the strength of the link between the nodes. We used CiteSpace (5.8. R3) to construct a dual-map overlay for journals, cluster analysis of co-cited references and keywords, and detection of references and keywords with strong citation bursts (Chen, 2004). The parameters of CiteSpace were set as follows: link retaining factor (LRF = 3), look back years (LBY = 5), e for top N (e = 1), time span (2009–2021), years per slice (1), links (strength: cosine, scope: within slices), selection criteria (g-index: k = 25), and minimum duration (MD = 2 for keywords; MD = 5 for references).

## 3 Results

### 3.1 Global Publication Trend

**FIGURE 1:**
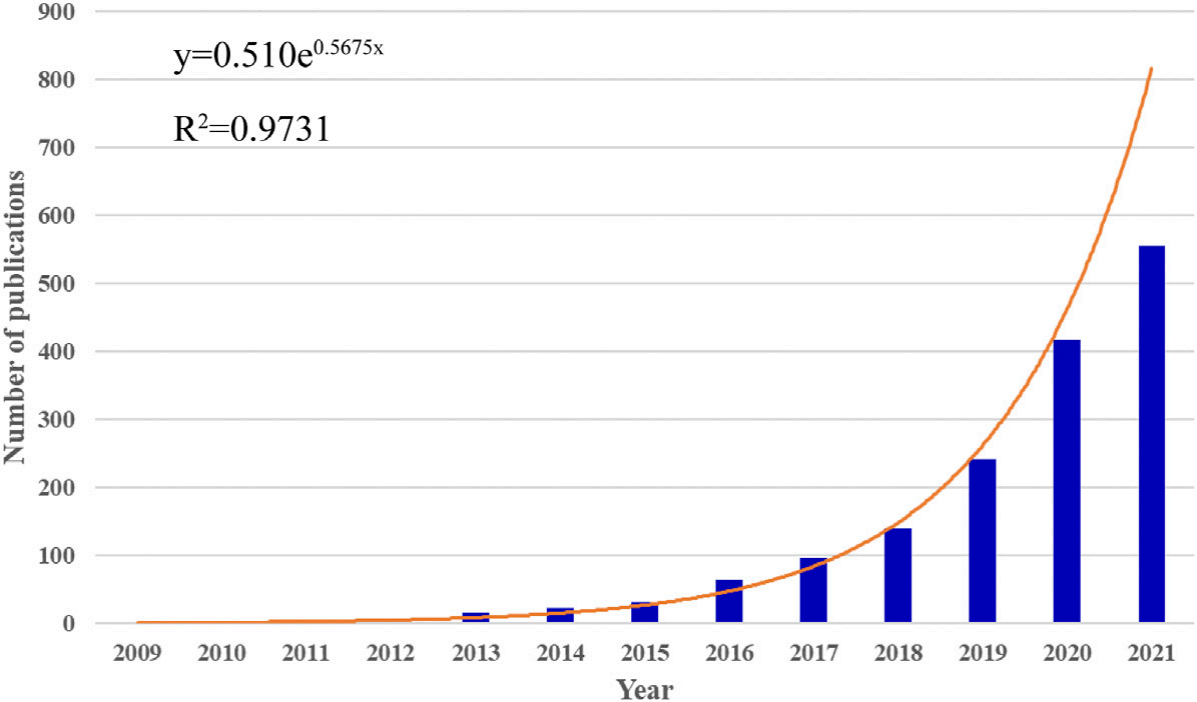
Global trends in publications on MSC-EVs. The bar graph displays the trends in the growth of publications worldwide from 2009 to 2021. The curve shows the model fitting curves of growth trends in publications.

In total, 1595 publications related to MSC-EVs were identified in the WoS from 2009 to 2021. Of these, 1351 (84.70%) were indexed as “article” and 244 (15.30%) were indexed as “review”. Between 2009 and 2021, the annual of publications grew rapidly from a few articles to over 500 articles (Figure 1). The annual growth trend is in line with the fitting curve y = 0.510e^0.5675x^ (*R* ^2^ = 0.9731) (Figure 1). This indicated that MSC-EVs have gradually attracted the attention of scholars, and may become a long-standing research hotspot.

### 3.2 Distribution of Countries and Institutions

**FIGURE 2:**
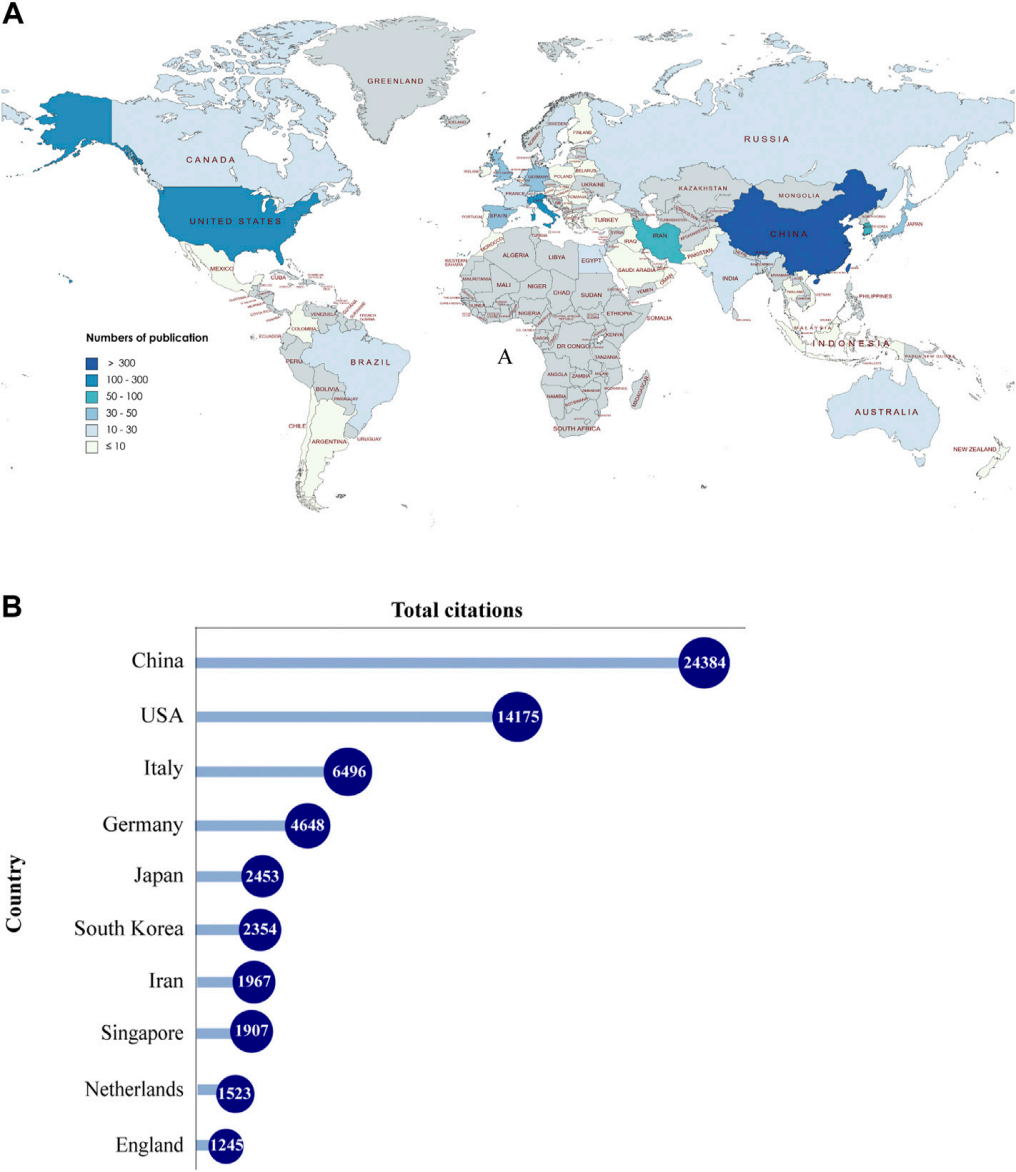
Countries contributing to MSC-EVs. **(A)** World map showing the distribution of countries in this field. **(B)** Top 10 countries with the most total citations.

**FIGURE 3:**
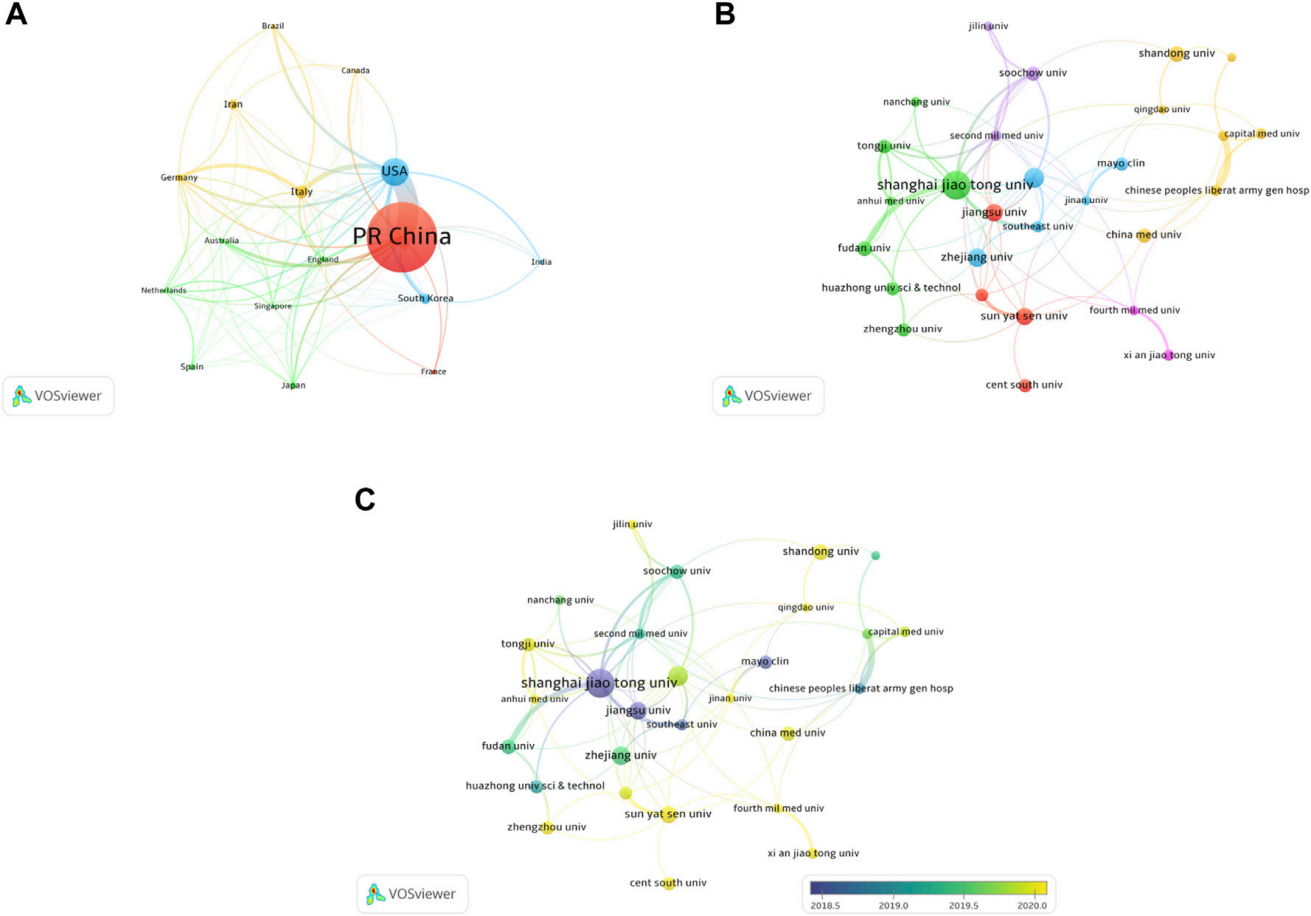
Co-authorship analysis of countries and institutions. **(A)** Network map of co-authorship between countries with more than 15 publications. **(B)** Network map of co-authorship between institutions with more than 15 publications. **(C)** Network map of co-authorship between institutions with more than 15 publications according to average publication year (blue: earlier, yellow: later).

A total of 55 countries around the world contributed to the research on MSC-EVs (Figure 2A). Of these, 17 countries (30.9%) are in Europe, 10 countries (18.2%) in Asia, and two countries (3.6%) each in South America and North America. As shown in Table 1 (Part A), China (899 publications, 56.36% of all articles) is the most productive country, followed by the United States (278, 17.43%), Italy (105, 6.58%), South Korea (80, 5.02%), and Iran (79, 4.95%). In terms of total citations, the top five countries are China (24,210 citations), the United States (14,615), Italy (6,485), Germany (4,652), and Japan (2,458) (Figure 2B). We analyzed the collaborative relationships between countries with more than 15 publications in the field, as shown in Figure 3A, where the size of the nodes represents the number of publications and the thickness of the connected lines represents the strength of the link. The United States has the highest total link strength (177 times) followed by China (132). The third to fifth places are Italy (64), Germany (59), and England (44).

**TABLE 1:** The top 10 countries and institutions with the most publications in the field of MSC-EVs.

Rank	Part A			Part B	
	Countries	Number of Publications	Number of Publications per 10 Million Population	Institutions	Number of Publications
1	China	899	6.37	Shanghai Jiao Tong University (China)	79
2	United States	278	8.39	Nanjing Medical University (China)	43
3	Italy	105	17.66	Zhejiang University (China)	39
4	South Korea	80	15.43	Central South University (China)	37
5	Iran	79	9.41	Jiangsu University (China)	37
6	Germany	50	6.01	University Of Turin (Italy)	36
7	Japan	45	3.58	Zhengzhou University (China)	36
8	Spain	41	8.66	Sun Yat Sen University (China)	35
9	England	35	5.21	University Of California System (United States)	34
10	France	30	4.45	Shandong University (China)	32

A total of 1692 institutions are involved in this field. The top 10 institutions with the most publications are listed in Table 1 (Part B). Shanghai Jiao Tong University (79 publications, 4.95% of all articles) contributed the maximum number of publications, followed by Nanjing Medical University (43, 2.70%), Zhejiang University (39, 2.45%), Central South University (37, 2.32%), and Jiangsu University (37, 2.32%). We analyzed the collaborations of 35 institutions with more than 15 publications. We excluded seven unrelated items and revealed the co-authorship of 28 institutions (Figure 3B). The five institutions with the highest total link strength were Shanghai Jiao Tong University (total link strength = 32 times), Second Military Medical University (23), Nanjing Medical University (21), Soochow University (18), and Tongji University (17). Figure 3C shows the average publication year of the 28 institutions above. The majority of institutions published papers after 2019, with greener or yellower colors. The institution with the earliest average year of publication is Jiangsu University (2018.25), and the institution with the latest average year of publication is Central South University (2020.64).

### 3.3 Analysis of Journals and Research Areas

**FIGURE 4:**
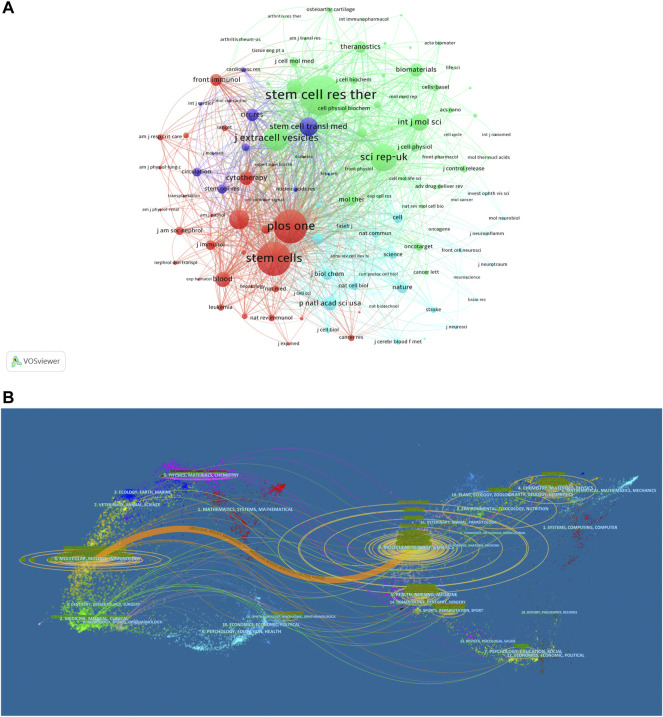
Articles published in different journals on MSC-EVs. **(A)** Network map of journals that were co-cited in more than 150 publications. **(B)** The dual-map overlay of journals related to MSC-EVs.

Since 2009, a total of 1595 articles were published in 437 journals. The top 10 journals with the most publications are listed in Table 2. Stem cell research & therapy (147 publications, 9.22% of all articles) had the most publications, followed by International journal of molecular sciences (44, 2.76%), and Stem cells international (43, 2.70%). We analyzed a total of 120 journals that were co-cited more than 150 times (Figure 4A). Table 2 shows the top 10 co-cited journals that published related articles. Top of the list is still Stem cell research & therapy (2707 citations), followed by Stem cells (2309), and Plos one (2274).

**TABLE 2:** The top 10 most active journals and co-cited journals.

Rank	Top Journals	Records (n)	2020 IF	2020 JCR	Co-Cited Journals	Citations (n)	2020 IF	2020 JCR
1	Stem cell research & therapy	147	6.832	Q1	Stem cell research & therapy	2707	6.832	Q1
2	International journal of molecular sciences	44	4.101	Q2	Stem cells	2309	6.277	Q1
3	Stem cells international	43	5.443	Q2	Plos one	2274	3.24	Q2
4	Frontiers in cell and developmental biology	35	6.684	Q1	Scientific reports	1788	4.38	Q1
5	Scientific reports	34	4.38	Q1	Journal of extracellular vesicles	1655	25.841	Q1
6	Journal of cellular and molecular medicine	33	5.31	Q2	Stem cells and development	1320	3.272	Q2
7	Cells	29	6.6	Q2	Stem cells translational medicine	1217	6.94	Q1
8	Plos one	26	3.24	Q2	International journal of molecular sciences	1144	4.101	Q2
9	Aging-us	24	5.682	Q1	Stem cells international	1017	5.443	Q2
10	Life sciences	24	5.037	Q1	Blood	973	23.629	Q1

IF, impact factor; JCR, journal citation reports; Q, quartile in category.

Overall, the identified publications are grouped into 63 research areas. As shown in Table 3, the most representative research area was Cell Biology (672 records, 42.13% of all articles), followed by Research Experimental Medicine (406, 25.46%), and Biochemistry Molecular Biology (185, 11.60%). In addition, a dual-map overlay of journals was used to analyze the association of subject categories between citing and cited journals. The spline wave from left to right describes the citation path, and this interaction illustrates the linkage of different research areas. Only one critical citation path marked in orange indicates that papers published in the journals in the area of Molecular/Biology/Immunology usually cited papers published in Molecular/Biology/Genetics journals (Figure 4B).

**TABLE 3:** The top 10 well-represented research areas.

Rank	Research Areas	Records (n)	% (of 1595)
1	Cell Biology	672	42.13
2	Research Experimental Medicine	406	25.46
3	Biochemistry Molecular Biology	185	11.60
4	Science Technology Other Topics	149	9.34
5	Pharmacology Pharmacy	131	8.21
6	Biotechnology Applied Microbiology	116	7.27
7	Oncology	109	6.83
8	Chemistry	87	5.46
9	Materials Science	83	5.20
10	Engineering	71	4.45

### 3.4 Analysis of Authors

The top 12 authors with the most publications and most citations are shown in Table 4. Camussi G, from the University of Torino, is the most productive author (21 articles), followed by Eirin A, from Mayo Clinica (19), and Lerman LO, from Mayo Clinica (16). As to citations in this field, Camussi G was ranked first (3379 citations) as well, followed by Bruno S, from the University of Torino (3,368), and Ciro Tetta, from Unicyte Srl (2,776). Notably, Camussi G had the highest number of publications and citations, indicating that he is probably at present the most active scholar and has made significant contributions to the development of the field.

**TABLE 4:** The top 12 authors with the most publications and citations on MSC-EVs research.

Rank	Highly Published Authors	Country	Number of Publications	Highly Cited Authors	Country	Total Citations (n)
1	Camussi G	Italy	21	Camussi G	Italy	3379
2	Eirin A	United States	19	Bruno S	Italy	3368
3	Lerman LO	United States	16	Tetta C	Italy	2776
4	Qian H	China	15	Deregibus MC	Italy	2620
5	Bruno S	Italy	15	Qian H	China	2052
6	Liu W	China	15	Xu W	China	2051
7	Xu W	China	14	Collino F	Italy	1979
8	Giebel B	Germany	13	Chopp M	United States	1614
9	Orfei CP	Italy	11	Yan Y	China	1508
10	Ragni E	Italy	11	Zhu W	China	1497
11	De Girolamo L	Italy	11	Lai RC	Singapore	1486
12	Wang Z	China	11	Grange C	Italy	1448

**FIGURE 5:**
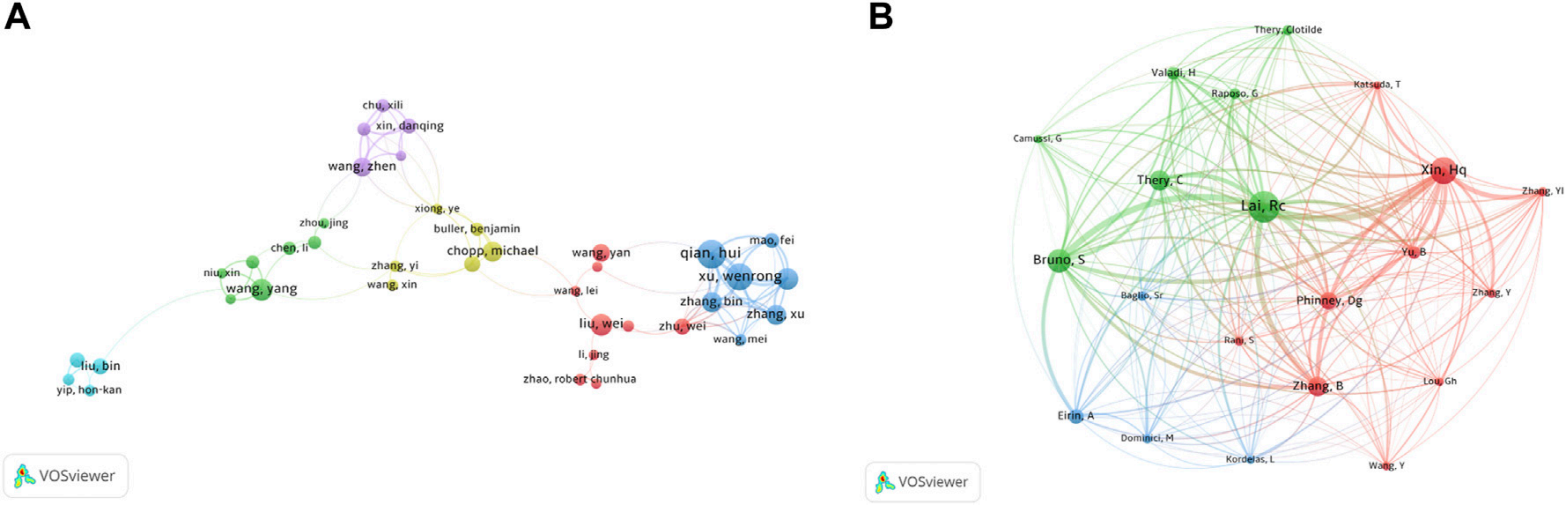
Analysis of authors. **(A)** Network map of co-authorship between authors with more than five publications. **(B)** Network map of co-cited authors with more than 150 citations.

We detected a total of 102 authors who co-authored more than five publications. Excluding the 64 items that were not connected, collaborations of 38 authors were shown (Figure 5A). The five authors with the highest total link strength were Qian H (total link strength = 74 times), Xu W (74), Yan Y (63), Zhang X (53), and Zhang B (49). Figure 5B showed the co-citation relationships for a total of 21 authors with more than 150 citations. In first place was Lai RC, with 601 citations, followed by Xin HQ (516), Bruno S (453), Théry C (390), and Zhang B (373).

### 3.5 Citation and Co-Citation Analyses

**FIGURE 6:**
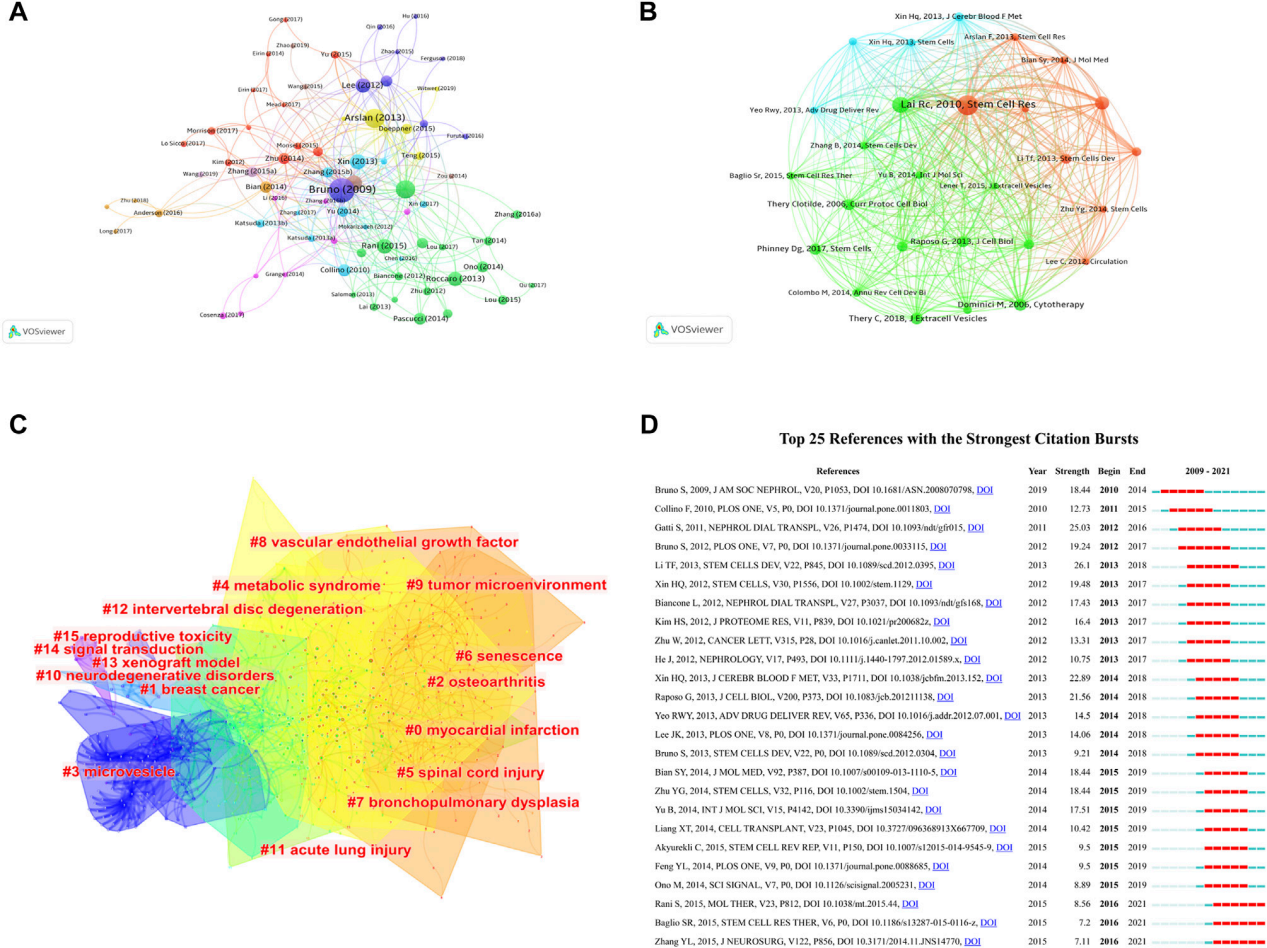
Mapping of documents and references in studies on MSC-EVs. **(A)** Network map of citation analysis of documents with more than 150 citations. **(B)** Network map of co-citation analysis of references with more than 50 citations. **(C)** Clustering analysis of the co-citation network based on CiteSpace. **(D)** Top 25 references with strongest citation bursts based on CiteSpace.

A total of 76 articles in this field have more than 150 citations (Figure 6A). The top ten most cited documents are shown in Table 5. There were 859 citations for “Mesenchymal stem cell-derived microvesicles protect against acute tubular injury”, followed by “Mesenchymal stem cell-derived exosomes increase ATP levels, decrease oxidative stress and activate PI3K/Akt pathway to enhance myocardial viability and prevent adverse remodeling after myocardial ischemia/reperfusion injury”, with 666 citations. The third-ranked article with the largest number of citations was “Concise Review: MSC-Derived Exosomes for Cell-Free Therapy”, with 642 citations.

**TABLE 5:** The top 10 documents with the most citations in the field of MSC-EVs.

Rank	Title	Corresponding Author	Journal	2020 IF	Publication year	Total citations (n)
1	Mesenchymal stem cell-derived microvesicles protect against acute tubular injury	Camussi G	Journal of the american society of nephrology	10.121	2009	859
2	Mesenchymal stem cell-derived exosomes increase ATP levels, decrease oxidative stress and activate PI3K/Akt pathway to enhance myocardial viability and prevent adverse remodeling after myocardial ischemia/reperfusion injury	Arslan F	Stem cell research	2.02	2013	666
3	Concise Review: MSC-Derived Exosomes for Cell-Free Therapy	Phinney DG	Stem cells	6.277	2017	642
4	Microvesicles derived from human adult mesenchymal stem cells protect against ischaemia–reperfusion-induced acute and chronic kidney injury	Camussi G	Nephrology dialysis transplantation	5.992	2011	540
5	Systemic administration of exosomes released from mesenchymal stromal cells promote functional recovery and neurovascular plasticity after stroke in rats	Chopp M	Journal of cerebral blood flow and metabolism	6.2	2013	526
6	Mesenchymal Stem Cell-derived Extracellular Vesicles: Toward Cell-free Therapeutic Applications	Rani S	Molecular therapy	11.454	2015	519
7	BM mesenchymal stromal cell-derived exosomes facilitate multiple myeloma progression	Ghobrial IM	Journal of clinical investigation	14.808	2013	508
8	Exosomes mediate the cytoprotective action of mesenchymal stromal cells on hypoxia-induced pulmonary hypertension	Kourembanas S	Circulation	29.69	2012	503
9	Microvesicles derived from adult human bone marrow and tissue specific mesenchymal stem cells shuttle selected pattern of miRNAs	Collino F	Plos one	3.24	2010	437
10	Paclitaxel is incorporated by mesenchymal stromal cells and released in exosomes that inhibit *in vitro* tumor growth: a new approach for drug delivery	Pessina A	Journal of controlled release	9.776	2014	420

IF, impact factor.

In total, 27 references, co-cited in more than 50 citations, were analyzed by VOSviewer (Figure 6B). Table 6 lists the top ten references with the highest citations. The top five references with the largest number of citations were by Lim SK (2010; 301 citations), Lötvall JO (2007; 239 citations), Camussi G (2009; 199 citations), Dominici M, (2006; 181 citations), and Théry C (2018; 177 citations). The co-cited references were then clustered based on indexing terms. As shown in Figure 6C, the co-cited references were clustered into 16 major clusters: myocardial infarction, breast cancer, osteoarthritis, microvesicle, metabolic syndrome, spinal cord injury, senescence, bronchopulmonary dysplasia, vascular endothelial growth factor, tumor microenvironment, neurodegenerative disorders, acute lung injury, intervertebral disc degeneration, xenograft model, signal transduction, and reproductive toxicity.

**TABLE 6:** The top ten co-citation analysis of cited reference on MSC-EVs.

Rank	Title	Corresponding Author	Journal	2020 IF	Publication year	Total citations (n)
1	Exosome secreted by MSC reduces myocardial ischemia/reperfusion injury	Lim SK	Stem cell research	2.02	2010	301
2	Exosome-mediated transfer of mRNAs and microRNAs is a novel mechanism of genetic exchange between cells	Lötvall JO	Nature cell biology	28.824	2007	239
3	Mesenchymal stem cell-derived microvesicles protect against acute tubular injury	Camussi G	Journal of the american society of nephrology	10.121	2009	199
4	Minimal criteria for defining multipotent mesenchymal stromal cells. The International Society for Cellular Therapy position statement	Dominici M	Cytotherapy	5.414	2006	181
5	Minimal information for studies of extracellular vesicles 2018 (MISEV 2018): a position statement of the International Society for Extracellular Vesicles and update of the MISEV2014 guidelines	Théry C	Journal of extracellular vesicles	25.841	2018	177
6	Isolation and characterization of exosomes from cell culture supernatants and biological fluids	Théry C	Current protocols in cell biology^a^	NA	2006	171
7	Concise Review: MSC-Derived Exosomes for Cell-Free Therapy	Phinney DG	Stem cells	6.277	2017	170
8	Extracellular vesicles: exosomes, microvesicles, and friends	Raposo G	Journal of cell biology	10.539	2013	161
9	Mesenchymal Stem Cell-derived Extracellular Vesicles: Toward Cell-free Therapeutic Applications	Rani S	Molecular therapy	11.454	2015	150
10	MSC-derived exosomes: a novel tool to treat therapy-refractory graft-versus-host disease	Kordelas L	Leukemia	11.528	2014	145

IF, impact factor.

a *Current protocols in cell biology* was not included in the 2020 Journal Citation Reports.

The top 25 references with the strongest citation bursts are presented in Figure 6D, among which, the article titled “Exosomes derived from human umbilical cord mesenchymal stem cells alleviate liver fibrosis”, published in 2013, ranked first (strength = 26.1). Moreover, the citation bursts of articles published by Rani S, Baglio SR, and Zhang YL all lasted from 2016 to 2021.

### 3.6 Co-Occurrence Analysis of Keywords

**FIGURE 7:**
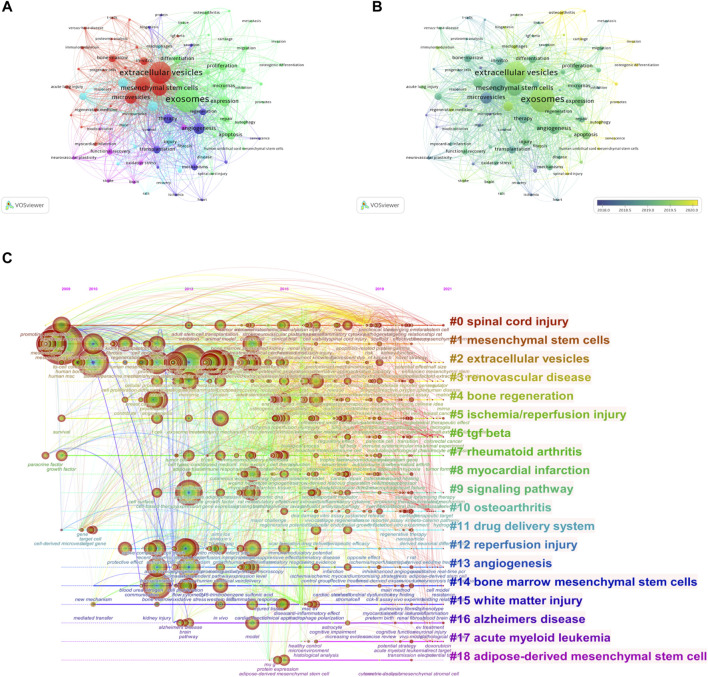
Mapping of keywords in studies on MSC-EVs. **(A)** Network visualization of keywords. **(B)** Distribution of keywords according to average publication year (blue: earlier, yellow: later). **(C)** Keyword timeline visualization from 2009 to 2021.

VOSviewer was employed to analyze the keywords that occurred more than 25 times in all publications included. We obtained a total of 88 keywords, of which the five with the highest frequency were exosomes (787 times), extracellular vesicles (659), mesenchymal stem cells (374), stromal cells (370), and microvesicles 258) (Figure 7A). The average publication year of the identified keywords is indicated in the overlay visualization (Figure 7B). The chronological order is presented from dark blue to bright yellow. The majority of the keywords were published during 2019, while small extracellular vesicles, cartilage, chondrocytes, and senescence were relatively new keywords that emerged after 2020.

The time dynamic evolution of keywords clusters is presented in Figure 7C. In total, 19 clusters were identified, namely, spinal cord injury, mesenchymal stem cells, extracellular vesicles, renovascular disease, bone regeneration, ischemia/reperfusion injury, TGF β, rheumatoid arthritis, myocardial infarction, signaling pathway, osteoarthritis, drug delivery system, reperfusion injury, angiogenesis, bone marrow mesenchymal stem cells, white matter injury, Alzheimer’s disease, acute myeloid leukemia, and adipose-derived mesenchymal stem cell. The average year of appearance for TGF-β was most recent, 2020, and the average year of appearance for osteoarthritis and acute myeloid leukemia was 2019.

**FIGURE 8:**
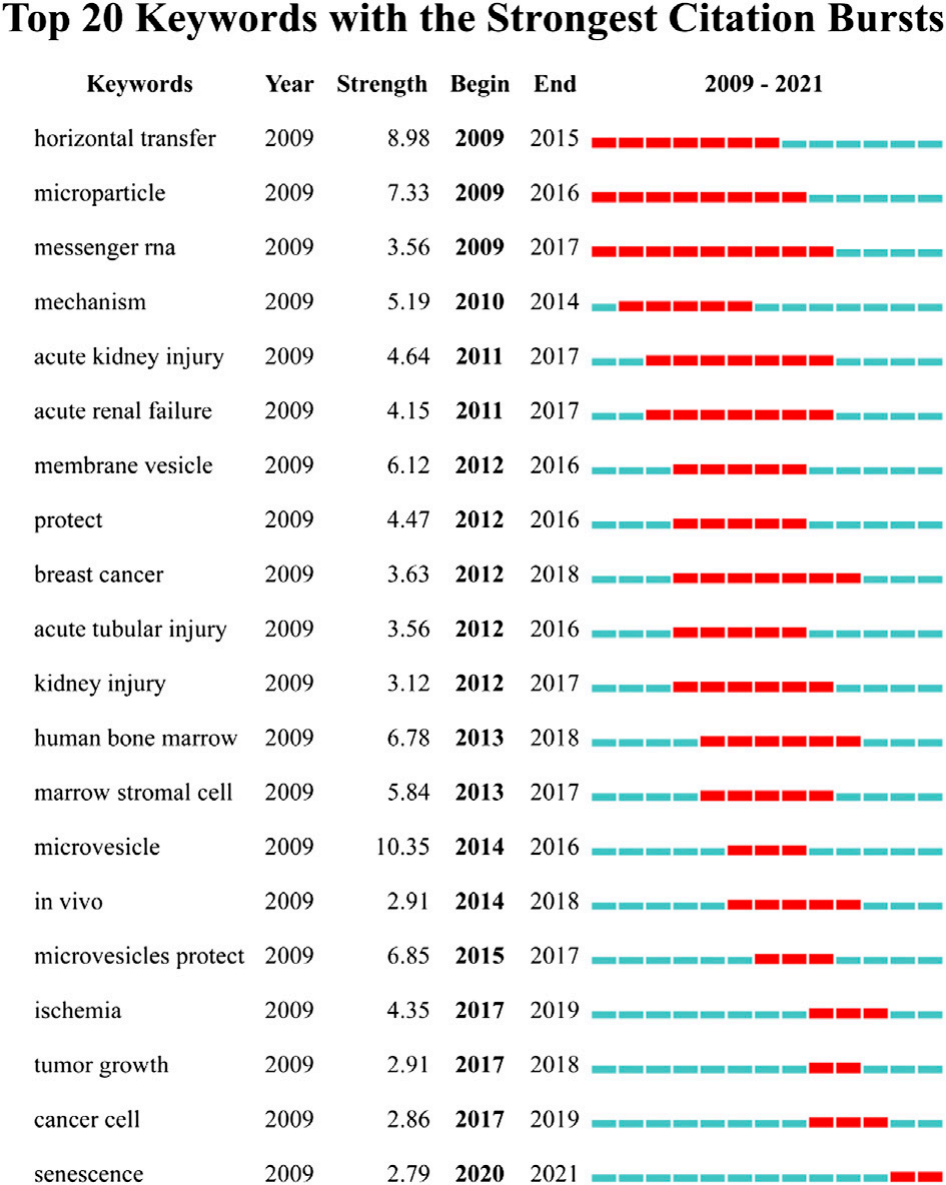
Top 20 keywords with the strongest citation bursts based on CiteSpace.

We detected the burst of keywords based on CiteSpace’s algorithm of burst detection, where the minimum duration of the burst was set to 2 years. The discontinuous blue lines represent the timeline, specifically, each small blue rectangle represents 1 year, and the red part in the timeline represents the burst duration of the keyword. The top 20 keywords with the highest burst strength are shown in Figure 8. The most intense keyword was microvesicle (strength = 10.35), followed by horizontal transfer (8.98) and microparticle (7.33). The keyword with the longest burst time was messenger RNA, which lasted 9 years from 2009 to 2017. More meaningfully, the keyword “senescence” had outbreak citations most recently (2020-2021), which implied that the research on the linkage between MSC-EVs and aging might be research hotspots in the future.

## 4 Discussion

In this study, we performed a bibliometric analysis of the literature published in the field of MSC-EVs from 2009 to 2021 based on visual management software. The temporal and spatial distribution of the literature, the contributions of countries, institutions, journals, authors, keywords, research areas, and future research hotspots were analyzed to clarify the evolutionary process and the changing in research focus and to guide researchers.

### 4.1 Overview of the Development of MSC-EVs

The earliest article that fits the theme of this study was published in 2009 by Bruno S et al. (Bruno et al., 2009). Since 2018, annual publications began to increase rapidly, with more than 500 publications in 2021, and articles in the past 3 years accounted for 76.11% of all identified articles. All these suggest that the research on MSC-EVs is developing rapidly as an emerging field. Furthermore, most of the top 10 most active journals and co-cited journals have impact factors above 5, indicating that the research in this field has relatively high academic value and the research findings are widely recognized. Stem cell research & therapy, which ranked first in both the number of publications and citations, reaching the top 1/4 (Q1) in the categories of Cell & Tissue Engineering, Cell Biology, and Medicine, Research & Experimental.

In terms of national contributions, China had the largest number of publications and citations in the world, indicating that China has become the most significant contributor to MSC-EVs research. We further analyzed the citations on average among countries with ≥50 publications and observed that Germany, Italy, United States were the top three countries with 92.96, 61.87, and 50.99 citations, respectively. Additionally, the United States played a leading role in this field, as it was far ahead of other countries in terms of centrality (0.52) and strength of collaboration (177). These findings suggested that the number of publications did not fully represent academic influence, and countries should encourage original creative discoveries and technological innovations beyond mere imitations and additions of previous breakthroughs. Ranking the academic achievements of research institutions, eight of the top 10 institutions came from China. The University of Turin in Italy had the most average citations (81.39) and an earlier publish year (2016.52).

### 4.2 Influential Authors and Studies in the MSC-EVs Research

Camussi G has published 21 papers on MSC-EVs, with a total of 3379 citations, and is the most productive and most cited author. From 2009 to 2021, Camussi G had publications every year except 2015 and could be considered a pioneer in the field. Camussi G mainly focused on the function of MSC-EVs in the repair of tissue damage and the key roles of various RNAs therein. In 2010, it was proposed that ribonucleoproteins involved in mRNAs and miRNAs transport, processing, and stabilization were present in MSC-EVs and that miRNAs highly expressed in extracellular vesicles might be involved in multi-organ development, cell survival, and differentiation (Collino et al., 2010). Camussi G was also devoted to the effect of MSC-EVs on renal injury, liver fibrosis, diabetes and its complications (wound ulcers), ischemia/reperfusion injury, brain injury, and tumors (Bruno et al., 2009; Bruno et al., 2014; Favaro et al., 2016; Lonati et al., 2019; Sisa et al., 2019; Chiabotto et al., 2021; Pomatto et al., 2021). Furthermore, Ratajczak MZ, Ratajczak J, Quesenberry PJ, Aliotta JM, and Lim SK *et al.* also laid a strong foundation for the pioneering field of MSC-EVs. In 2006, the Ratajczak laboratory from the University of Louisville demonstrated that embryonic stem cell (ES)-derived microvesicles can mediate the expansion and upregulation of pluripotency in hematopoietic progenitor cells through horizontal transfer of ES-derived mRNA (Ratajczak et al., 2006). The Rhode Island Hospital research team, to which Quesenberry PJ and Aliotta JM belonged, began to focus on the impact of niche on stem cell fate in 2007 (Aliotta et al., 2012) and proposed that microvesicles in the niche could affect the differentiation of marrow stem cells (Quesenberry and Aliotta, 2008; Quesenberry et al., 2015). And they had cooperated with Camussi G to conduct research on MSC-EVs since 2014 (Lindoso et al., 2014). Professor Lim SK from Nanyang Technological University analyzed the conditioned medium of ES-derived MSCs. The results suggested that MSCs could secrete abundant proteins and microparticles enriched for pre-miRNA, and these gene products were predicted to drive three major groups of biological processes: metabolism, defense response, and tissue differentiation (Sze et al., 2007; Chen et al., 2009). In brief, the early contributions of the above teams all provided a solid foundation for the shift from paracrine to EVs in the study of MSCs.

Notably, collaboration among authors in this field is not close. Of the 102 authors with more than five publications, only 38 authors (approximately 1/3) could construct collaborative networks. Furthermore, in the near-linear cooperation map, almost all scholars are Chinese, and the cooperation is mostly confined within the research team. Scholars from different countries should strengthen cooperation, share advantageous platforms, exchange research progress, and form complementary advantages to achieve technological innovation and breakthroughs for the clinical translation of MSC-EVs.

The citation analysis of the publications and the co-citation analysis of references suggest the current research focuses on the therapeutic role of MSC-EVs in tissue repair and functional remodeling. In 2009, the study by Bruno S et al., with the most citations, reported that MSC-derived microvesicles initiate a proliferative program by transferring mRNA to residual renal tubular cells, thereby rescuing acute renal tubular injury (Bruno et al., 2009). The second most cited was the publication by Arslan F et al. which reported that infusion of MSC-derived exosomes into mice ischemic myocardium through the aorta could prevent ventricular dilatation and improve cardiac function (Arslan et al., 2013). Exosomes can improve energy imbalance, oxidative stress, and inflammatory responses induced by ischemia/reperfusion. Among the references with high co-citations, more attention lay on the extraction and identification criteria of EVs, in addition to the therapeutic potential of MSC-EVs in traumatic diseases. Théry C proposed a specific protocol for the isolation and characterization of exosomes from cell culture supernatants and biological fluids in 2006 (Dominici et al., 2006). In 2018, the International Extracellular Vesicle Society (ISEV) updated the Minimal Information for Studies of Extracellular Vesicles guidelines with the most important statement that exhaustive reporting is required when conferring specific functions on EVs, and when describing the delicate activities of EVs careful interpretation is necessary, given that the molecular mechanisms of its biogenesis and release remain to be explored (Théry et al., 2018). Notably, the guidelines or statements issued by the ISEV and the International Society for Cellular and Gene Therapies (ISCT) are recognized as the standard that MSC-EVs research should follow. The research group led by Rohde E, Lim SK, and Giebel B, respectively, contributed important knowledge to the field of MSC-EVs, and they participated in drafting several statements or reviews as members of ISEV and ICTG, including defining mesenchymal stromal cell (MSC)-derived small extracellular vesicles for therapeutic applications (Witwer et al., 2019), applying extracellular vesicles based therapeutics in clinical trials - an ISEV position paper (Lener et al., 2015), developing Best-Practice Models for the Therapeutic Use of Extracellular Vesicles (Reiner et al., 2017), and ISEV and ISCT statement on extracellular vesicles from mesenchymal stromal cells and other cells: considerations for potential therapeutic agents to suppress coronavirus disease-19 (Börger et al., 2020). Moreover, studies on RNAs wrapped in EVs were also the focus of high co-cited references and strong citation bursts in recent years. Valadi H et al. defined an exosomal shuttle RNA, which mainly includes mRNAs and miRNAs (Valadi et al., 2007). Most of the mRNAs were not present in the cytoplasm of the donor cell but could be delivered to the recipient cell and translated into new proteins to function. In an analysis of exosomes from BMSCs and adipose-derived MSCs (ADSCs), Baglio SR et al. demonstrated that stemness and multipotent of MSCs affected the composition of exosomes and that the RNA composition of exosomes was not the same as those of MSCs, with exosomes being able to selectively incorporate specific miRNAs (Baglio et al., 2015).

### 4.3 Research Hotspots and Future Trends

As shown in Table 5, MSC-EVs involved a wide range of research areas, forming a multidisciplinary convergence pattern dominated by cell biology. In particular, the emergence of materials science and engineering in the study of MSC-EVs strengthened the development of their therapeutic role. Different from MSCs, EVs lack the ability to self-renew and proliferate, hence the duration of activity maintenance is short. Yang et al. developed an injectable Diels–Alder crosslinked hyaluronic acid/PEG hydrogel, which achieved the sustained release of MSC-EVs in the joint cavity through degradation control, and hence, improved the therapeutic effect of osteoarthritis (Yang et al., 2021). Nanohydroxyapatite/poly-ε-caprolactone scaffolds with hyaluronic acid hydrogels mixed with MSC-derived exosomes as a sustained-release system could promote angiogenesis and osteogenesis (Zhang Y. et al., 2021). Engineered EVs are obtained by genetic modification of donor cells or chemical modification of EVs for targeted delivery, increasing local concentrations at diseased sites, reducing toxicity and side effects, and maximizing therapeutic efficacy (Liang et al., 2021). Tsai HI et al. modified MSCs to obtain engineered EVs expressing FGL1 and PD-L1 on the membrane surface, which could target and bind to LAG-3 and PD-1 on the surface of T cells, thereby inhibiting T cell activation, and inducing immune tolerance in a heart allograft model (Tsai et al., 2022). The multidisciplinary combination helped to broaden the research horizon, overcome the research challenges, and lay the foundation for clinical translation in the field of MSC-EVs.

As shown in Figure 7, we can note that the therapeutic effects of MSC-EVs involved multi-system diseases, including neurological diseases, respiratory diseases, cardiovascular diseases, liver and kidney injuries, endocrine diseases, bone and joint diseases, reproductive system diseases, immune diseases, and cancer. The two diseases that appeared most frequently in the keywords were osteoarthritis and cancer. Osteoarthritis is a degenerative disease characterized by damage to cartilage, and cartilage degeneration is the most basic pathological change. MSC-derived exosomes could promote the proliferation of chondrocytes and inhibit their apoptosis to regenerate cartilage (Liu et al., 2018). Coordination the local inflammatory and regeneration microenvironment is crucial to the treatment of OA (Zhao et al., 2020). Zhang *et al.* demonstrated that MSC-derived exosomes attenuated inflammation and suppressed cartilage degeneration in the early stage, and subsequently restore matrix homeostasis for joint recovery and regeneration (Zhang et al., 2019). MSC-EVs may both promote tumor development and act as anti-tumor agents. MSCs in the tumor microenvironment could be reprogrammed by exosomes secreted by tumor cells, and MSC-derived exosomes then horizontally transferred information to neighboring cells, transforming the cellular milieu into one supportive of the tumor survival (Whiteside, 2017). Different MSC-EVs had diverse effects on specific tumors. For example, Guo *et al.* discovered that EVs derived from human umbilical cord-derived MSCs (hUCMSCs) promoted proliferation, migration, and invasion of lung cancer and accelerated tumor progression (Guo et al., 2021). Nevertheless, another study on the treatment of lung cancer with BMSCs-derived EVs suggested that EVs-derived let-7i could inhibit tumor proliferation and metastasis (Liu et al., 2021). Such differences might arise from the heterogeneity of MSCs, differences in EVs cargo, the diversity of malignancies’ origin, and inconsistencies in experimental conditions (Weng et al., 2021). Using bioengineered EVs as delivery vehicles appears to be a more promising therapeutic approach in cancer, as they can transfer desired cargoes and confer enhanced targeting specificity (Walker et al., 2019).

From the co-occurrence of keywords, it can be observed that MSC-EVs come from a wide range of sources, among which BMSCs, ADSCs, and hUCMSCs are the main research candidates. Given the different types and abundance of proteins and RNAs in EVs derived from different MSCs, the selection of tissue-specific MSCs should be based on the pathological characteristics of diseases. According to available studies, ADSCs-derived EVs contained high levels of pro-angiogenic factors, BMSCs-derived EVs enriched with pro-differentiation and chemotactic proteins, and EVs secreted by hUCMSCs exhibited strong anti-inflammatory effects (Gorgun et al., 2021; Gupta et al., 2022). Therefore, further studies are warranted to elucidate the specific effects of different sources of MSC-EVs on various physiological processes and their strength, to guide application in disease therapy. Moreover, the safety of MSC-EVs in therapy should also be considered. The haemocompatibility of MSCs products is a key factor affecting patients’ safety. One of the major risks of intravascular MSCs therapeutics is that the highly procoagulant tissue factor (TF) carried by MSCs may adversely trigger the instant blood-mediated inflammatory reaction. In turn, TF can be incorporated into EVs, introducing a potential risk to the MSC-EVs therapy (Moll et al., 2019; Ringdén et al., 2022). Compared with ADSCs and perinatal tissue-derived MSCs, BMSCs displayed lower levels of TF (Moll et al., 2019) and might be relatively safer. However, our understanding is not comprehensive enough, and further studies are needed to compare the safety of MSC-EVs from different sources and their possible risk factors.

Keyword burst detection indicated that senescence has been explosively cited since 2020 and is likely to be an emerging research hotspot. For one thing, the senescence of MSCs could affect the secretion, contents, and biological functions of EVs (Boulestreau et al., 2020). Increased secretion of EVs by senescent MSCs appears to remove unnecessary, toxic, and misfolded molecules, thereby the altered cargos of EVs may affect their biological function (Ahmadi and Rezaie, 2021). BMSCs-derived EVs from aged mice could mediate age-related insulin resistance by targeting adipocytes, myocytes, and hepatocytes via high expression of miR-29b-3p in EVs (Su et al., 2019). In contrast to EVs isolated from young MSCs, senescent MSC-EVs failed to protect against the LPS-induced acute lung injury model in mice (Huang et al., 2019). The reason for this difference may be that several miRNAs associated with macrophage polarization in aged MSC-EVs differ from those in young MSC-EVs, affecting the switch of macrophages to an anti-inflammatory phenotype. In addition, one of the characteristics of senescent MSCs is the senescence-associated secretory phenotype (SASP) including pro-inflammatory cytokines, chemokines, growth factors, and proteases (Turinetto et al., 2016). EVs may also represent a non-canonical part of SASP that contributes to cancer progression by inhibiting the immunomodulatory function of MSCs (Boulestreau et al., 2020). For another, MSC-EVs can also play a role in preventing physiological aging. BMSCs-derived EVs from young mice were able to reduce cellular senescence, improve stem cell function and extend the life span of mice (Dorronsoro et al., 2021). MSC-EVs also exhibited therapeutic potential in a range of age-related diseases, including myocardial infarction (Charles et al., 2020), breast cancer (Sandiford et al., 2021), osteoarthritis (Zhang et al., 2019), neurodegenerative disorders (Cone et al., 2021), and intervertebral disc degeneration (Xia et al., 2019), as seen in the co-citation clustering analysis. The aging of the global population has led to a significant increase in the prevalence of age-related degenerative diseases, which not only negatively affects their quality of life but also imposes a significant burden on the healthcare system (Boulestreau et al., 2020). To solve this problem, an in-depth exploration of the role and mechanism of MSC-EVs in age-related diseases is urgently needed and necessary. Therefore, senescence-related topics might be the hotspot of future research on MSC-EVs.

### 4.4 Strengths and Limitations

We conducted a comprehensive bibliometric analysis of the research on MSC-EVs to introduce the research status, analyze the hotspots, and predict the research trend for the first time. In addition, a variety of tools, including R-bibliometrix, VOSviewer, and CiteSpace, were used to ensure the reliability and objectivity of the results. However, the present study still has several limitations. First, only the WoS database was used for relevant publication search, while the publications in other databases, such as PubMed, Embase, Scopus, and so on, might be omitted in this study, which may introduce the selection bias. Second, given that MSC-EVs is an emerging field of research, the latest studies published in high-quality journals might be overlooked in citation and co-citation analysis due to their low citations. Moreover, some publications related to MSC-EVs without a detailed definition of MSCs or EVs might be ignored. Finally, there is no uniform standard for parameter settings in Citespace, and hence, in the cluster and burst analysis, the outputs may vary slightly with different settings.

## 5 Conclusion

Using bibliometrics and visualization software, we summarized and analyzed the global research status, development trends, hotspots, and frontier themes of MSC-EVs. In recent years, the field of MSC-EVs has received great attention and grown rapidly. The keyword and co-citation clustering analysis indicated that current researches mainly focus on the therapeutic or supportive therapeutic efficacy of MSC-EVs in various diseases, such as cardiovascular diseases, neurodegenerative diseases, bone and cartilage diseases, and tumors. As well as the underlying mechanisms involved, including angiogenesis, improvement of the local microenvironment, and so on. Multidisciplinary collaboration is a trend in this field that could contribute to promoting the clinical application of MSC-EVs. The theme of linking senescence with MSC-EVs may be a frontier in the future.

## Data Availability

The original contributions presented in the study are included in the article/Supplementary Material, further inquiries can be directed to the corresponding author.
